# Breaking barriers: How transwomen meet their healthcare needs

**DOI:** 10.4102/phcfm.v16i1.4598

**Published:** 2024-06-29

**Authors:** Millicent Maoto, Burt Davis

**Affiliations:** 1 Africa Centre for HIV/Aids Management, Faculty of Economics and Management Sciences, Stellenbosch University, Stellenbosch, South Africa

**Keywords:** transgender women, stigma, HIV, alternative healthcare, hormone replacement therapy, misgendering, discrimination

## Abstract

**Background:**

Transgender women – individuals assigned male at birth but who identify as female – are disproportionately affected by, among others, human immunodeficiency virus (HIV), other sexually transmitted diseases (STIs) and mental health issues. Studies show that transgender women often encounter discrimination and stigma when seeking healthcare from health facilities.

**Aim:**

This study assessed the healthcare needs of transgender women, their experiences of the mainstream healthcare system and alternative strategies for navigating the healthcare system.

**Setting:**

The study was carried out in the City of Ekurhuleni Metropolitan Council in South Africa’s Gauteng province.

**Methods:**

A case study design was followed. Participants were purposively selected and included 10 transgender women aged 26–50. Individual semi-structured interviews were conducted over 2 months.

**Results:**

Participants expressed a need for hormone replacement therapy, HIV treatment and prevention and treatment for STIs. Experiences of participants within the healthcare system were predominantly negative, with instances of discrimination, stigma and privacy violations being commonplace. Alternative strategies to meet their healthcare needs included the use of self-medication, consulting traditional healers and utilising non-governmental organisations.

**Conclusion:**

There is an urgent need for equitable and inclusive health management of transgender women in South Africa.

**Contribution:**

This study provided a first look in a South African context into how and to what extent transwomen employ alternative healthcare strategies such as self-medication and utilising non-governmental organisations when faced with mainstream healthcare access barriers. The use of traditional doctors was identified as a novel, alternative strategy used by transwomen to access healthcare and treatment.

## Introduction

Transgender women – individuals assigned male at birth but who identify as female^1^ – are disproportionately affected by human immunodeficiency virus (HIV) and other sexually transmitted diseases (STIs).^2;3^ The World Health Organization reports that transgender women are 13 times more likely to be living with HIV compared to other reproductive-age populations.^4^ Moreover, there is a high prevalence of mental health issues among the transgender community.^5,6,7^ When accessing health services to address these and other health-related issues, transgender women must fit themselves into a binary gender system that only recognises and maintains male and female genders.^8^ As a result, transgender women consistently encounter discrimination and stigma when seeking healthcare from mainstream facilities, often from healthcare providers themselves.^9,10,11,12^ Transgender women in rural areas are often worse off, as they regularly face limited or no access to organisations providing gender-affirmative care.^12^

Many transgender women have resorted to developing alternative strategies to navigate the healthcare systems and fulfil their healthcare needs.^13,14^ Some transgender women resort to unofficial channels such as acquiring medicine without prescriptions from pharmacies, the black market or relying on assistance from friends to access healthcare.^12,13,15^ In more severe cases, instances of theft and fraud have been reported as means of accessing necessary healthcare services.^6^ Other transgender women have established support networks, connecting with individuals in similar situations to share experiences and provide guidance on accessing healthcare services that may be challenging to obtain.^12^ Those who have the means and access to the Internet utilise online resources, not only to gather healthcare information but also to connect with transgender communities globally.^12,16^ Others still choose to rather avoid or postpone seeking medical attention completely to circumvent facing these challenges.^17^

Clearly, transgender women battle to gain equitable access to healthcare services. To the best of our knowledge, little empirical research exists about the alternative strategies transwomen employ in South Africa to seek medical help. So far, research in South Africa has mainly focussed on the healthcare experiences of transgender women when accessing the mainstream healthcare system.^8,18^ This study aimed to provide a first look into how South African-based transwomen employ alternative healthcare strategies when faced with healthcare access barriers. At the same time, the study intended to offer further insights into how transgender women can be better accommodated in healthcare systems more generally.

To this end, we investigated how and to what extent transgender women utilise healthcare services in South Africa. We also wanted to identify the strategies this vulnerable key population employs to navigate healthcare systems. In light of the HIV prevalence among transgender women in South Africa ranging between 45% and 63%^19^, this study had a specific focus on how transwomen access HIV treatment and prevention services.

## Research methods and design

### Study design

A qualitative case study design was conducted with 10 transgender women, aged 26–50, who were purposively selected as participants for the study.

### Materials

Recruitment materials were disseminated to transgender women via the Ekurhuleni Pride Organisation Committee (EPOC) database so to create awareness about the study and attract potential participants. Individual semi-structured interviews were conducted over 2 months with each interview lasting between 30 min to an hour. The interview guide was developed in English. All interviews were audio recorded and transcribed verbatim for analysis.

### Participants

Participants were residents of the Ekurhuleni Metropolitan District in South Africa’s Gauteng province. As mentioned, recruitment efforts involved collaboration with EPOC, which facilitated the recruitment of eight participants. Individuals already participating in the study referred another two participants.

Educational backgrounds varied among participants: with six completing high school, three holding tertiary qualifications and one having an incomplete high school education. Employment status also varied: with four participants unemployed, two self-employed and the remainder formally employed. All participants identified as black and had previous interactions with mainstream healthcare facilities, making them purposively selected for the study.

### Procedure

Prospective participants who received the recruitment materials contacted the researcher directly to express interest in participating. Screening questionnaires were administered to assess participant eligibility based on criteria including age (18 and above), gender identity (identifying as female for at least 6 months), transgender identity, awareness of HIV status and willingness to disclose HIV status to the researcher. Prior to the interviews, written informed consent was obtained. Each participant was assigned a numerical identifier for data analysis and study report purposes. The interviews were conducted at participants’ homes to ensure comfort, privacy and a safe space.

### Measures

The interview guide covered the demographic details of the participants, as well as questions about:

- the healthcare needs of transgender women
- their access to healthcare and related experiences
- alternative healthcare solutions. The interview guide also included specific questions about HIV prevention, treatment and care. All questions were researcher designed.

### Data analyses

All the interviews were recorded using audio and later transcribed for analysis. The data analysis followed a six-step method outlined by Braun and Clarke.^20^ The authors utilised a descriptive and *in vivo* coding approach throughout the analysis.

To ensure the validity and reliability of the study, several criteria were considered.^21^ The criteria included credibility, dependability, transferability and confirmability. To achieve credibility, the authors used purposeful sampling and maintained close engagement with the data. Dependability was ensured through peer debriefing, where the findings were discussed with colleagues to validate interpretations. Transferability was addressed by including direct quotes from participants, enhancing the authenticity of the study’s findings. To maintain confirmability, the data gathered was cross-checked with the participants during the data collection.

### Ethical considerations

Ethical clearance to conduct this study was obtained from the Stellenbosch University Social, Behavioural and Education Research Ethics Committee (REC: SBER) (Project no.: 23522).

## Results

The analysis of the data revealed three primary themes and seven sub-themes (see Table 1).

**TABLE 1:** Summary of themes and sub-themes.

Themes	Sub-themes
Healthcare needs	The transitioning journey HIV status
Experiences of the healthcare system	Being treated as a human Respect for pronouns
Alternative healthcare strategies	Self-medication Non-governmental organisations (NGOs) Using traditional healers

### Theme 1: Healthcare needs

The experiences of the participants in relation to transitioning and fulfilling their HIV healthcare needs were shared as highlighted in the two sub-themes discussed in this section.

#### Sub-theme 1: The transitioning journey

All participants except one indicated that mainstream health facilities do not provide hormone replacement therapy (HRT), which they need to be able to fulfil their identity of being women. This aligns with prior research suggesting a widespread utilisation of hormones among transgender women. For instance, Rashiel et al.^22^ found in their research on hormone usage among transwomen on the West Coast of Malaysia that approximately 82% of transgender participants reported using hormones. Likewise, Smart et al.^23^ in their study examining the social determinants affecting the health, healthcare encounters and healthcare priorities of transgender women of colour in the southern United States, highlighted HRT as a prioritised healthcare requirement for transgender women.

Some participants who accessed HRT through non-governmental organisations (NGOs) and other public hospitals shared their frustration with the process. The process involved in obtaining gender-affirming care from specified state facilities or clinics is often cumbersome. This is evidenced by a study by Jeranji,^24^ which highlighted the lengthy waiting lists for gender-affirming care among disadvantaged transwomen in South Africa. As a result, some turned to the black market to access hormones, which resonates with research by Carosella et al.^25^ and Rashiel et al.^22^ in their studies on self-reported needs and health services utilisation among transgender women in Peru and Malaysia, respectively.

Among the negative health experiences related to taking hormones included that one must be under constant monitoring by the doctor and they experience problems when taking both hormones together with antiretroviral drugs (ARVs). As Participant 3 noted:

‘As a transitional woman who desires to grow breasts and change their male features; it would be nice for our general clinic to be able to supply our hormones to us.’ (Participant #3, 26 years old, make-up artist)

Moreover, participants reported that the challenges they experienced were not only at the structural level of the healthcare system’s deficiencies and personal interaction with doctors but also at a personal health level because of the risks associated with hormone use. This situation has also forced some participants to make the tough decision of halting their transition to prioritise their health.

#### Sub-theme 2: Human immunodeficiency virus status

The participants’ HIV healthcare needs were looked at from both the perspectives of transgender women who self-reported to be HIV positive and those who self-reported to be HIV negative. All participants irrespective of their HIV status expressed a need for access to HIV testing services, ARVs, pre-exposure-prophylaxis (PrEP), STI treatment, male and female condoms and lubricants.

Most of the participants reported that ARVs and PrEP at the mainstream facilities are available and accessible. About half of the participants also cited the importance of receiving emotional support as one of the healthcare needs of transgender women.

Participants also reported the importance of HIV education for all transgender women and specific to the LGBTQI+ community as a whole. Primary healthcare facilities often fail to provide tailored HIV education for transgender women and the LGBTQI+. This aligns with Sekoni et al.’s findings, which highlighted the lack of visibility of the LGBTQI+ community in primary healthcare setups concerning HIV prevention and education, based on their research on Hidden Populations in Nigeria. This omission is consistent with literature indicating the neglect of transgender populations in national HIV strategic plans in many countries with a high burden of HIV as noted by Samuel.^26^

Overall, there were strong similarities in awareness of risks and how to address them irrespective of HIV status.

### Theme 2: Experiences of the healthcare system

#### Sub-theme 1: Being treated as human

Most experiences of participants navigating the mainstream healthcare system were negative. They reported experiencing stigma and discrimination, being questioned by medical staff (i.e. staff were curious and lacking education about trans issues), being gossiped or humiliated by medical staff and lack of privacy. All participants reported having experienced ill-treatment at a mainstream healthcare facility. Some participants reported avoidance of healthcare attendance by transgender women altogether. The depth of effect of ill-treatment of transgender women appeared to hurt them deeply. As Participant 6 stated:

‘We want to be treated like humans because we are also human; treat us nicely address us as other human beings … Not to be made to feel like some other species; like we don’t belong on planet Earth.’ (Participant #6, 36 years old, social auxiliary worker)

However; some participants reported having positive experiences of accessing mainstream healthcare facilities. This was also echoed by Abreu et al.^27^ in their study looking into how Latino transgender women in Florida navigate the healthcare system. While these results suggested positive experiences among transgender women within the healthcare system, these instances mostly stemmed from personal interactions or connections with staff. As Participant 4 pointed out:

‘People are not the same; you do find nice nurses you know and there’s going to be that one nice nurse that we all know that if we go to sister so and so or sister that one we know that she is going to give you; you are going to get good services.’ (Participant #4, 35 years old, chef)

Seen as a whole, findings pointed to transgender women encountering major mistreatment within mainstream healthcare facilities. While Isano et al.^10^ examined healthcare experiences in Rwanda, De Santis et al.^9^ focussed on healthcare satisfaction in South Florida. Both studies found denial of services and pervasive stigma and discrimination towards transgender individuals. Similar findings were echoed by Luvuno et al.^8^ in South Africa and Mbeda et al.^28^ in sub-Saharan Africa, along with additional research by De Santis et al.^9^ All these studies reported a pattern of mistreatment and lack of privacy experienced by the transgender women population in healthcare settings.

Participants stated that the mistreatment by staff may stem from a lack of understanding, tolerance and education regarding the healthcare needs of transgender women. This aligns with findings from Frank et al.^29^ in the United States in their study focussing on the unmet healthcare needs of young transgender women and Abreu et al.^27^ These studies emphasised the importance of empowering healthcare providers with the necessary skills to address the needs of transgender women.

#### Sub-theme 2: Respect for pronouns

Almost all participants mentioned having experienced misgendering by healthcare employees from the reception to the actual nurses providing healthcare services. As Participant 3 stated:

‘I want them to simply know how to address me as she and her; because obviously of how I look basically. Before having to open forms and having to see that “Oh ok we see you are male here; what do you want us to address you as” that’s really making me feel uncomfortable; because you have to dissect how because you get addressed due to appearance compared to what my ID actually says.’ (Participant #3, 26 years old, make-up artist)

Several other studies also reported a disregard for transgender individuals’ preferred pronouns. For example, a study investigating transgender individuals’ experience of cancer screening in the United States highlighted a lack of respect for preferred pronouns.^30^ Similar findings were observed in a study examining HIV management services within primary healthcare facilities among the LGBTQI+ community in South Africa.^11^ Furthermore, a study conducted in South California on barriers to HIV PrEP uptake among Black and Hispanic transgender women also identified a failure to adhere to preferred pronouns.^31^ These studies collectively highlighted a broader issue of disrespect and insensitivity towards transgender identities within healthcare settings.

### Theme 3: Alternative healthcare strategies

#### Sub-theme 1: Self-medication

Almost all participants reported having self-medicated either at the advice of friends, utilising connections within the mainstream healthcare facilities or acquiring medicine without prescriptions from pharmacies.

Results showed that alternative strategies used by transgender women were mostly those that related to the prevention of HIV and reinfection. However, they seemed to require HRT most often. Hormone replacement therapy was reported to be almost impossible to access in public health facilities, as patients must have the financial means to consistently receive medication to ultimately transition. Alternative strategies to obtain HRT included accessing healthcare items from the black market.

These findings align with previous studies that also observed that transgender women often obtain medication through unconventional means such as online purchasing without a prescription, sharing among friends or even stealing.^6,10,25,32^ Obtaining medications from pharmacies without a prescription is consistent with findings reported by Sekoni et al.^12^ who found preferences by transgender women in utilising pharmacies to access medication. Seeking advice and recommendations from friends who have encountered similar health issues has also been documented by Augustaitis et al.^16^ in the United States. Their study on how online platforms assist transgender individuals in accessing health information revealed that seeking guidance from peers who have faced similar health challenges is prevalent within this community. Likewise, Sekoni et al.^12^ highlighted the significant role of network platforms in providing support and guidance regarding medication for transgender women.

#### Sub-theme 2: Accessing non-governmental organisations

About half of the participants reported utilising NGOs or outreach organisations to access HIV and other services. Non-governmental organisations were found to play an important role in providing support and other services to transgender women. Transgender women preferred the NGOs because they felt respected and comfortable and received quality care. Participant 2 described the role of NGOs as follows:

‘If you don’t need admission there are so many ways of going around and not go to the government because there’s NGOs with open clinics that specifically focus on us and our own issues. I find that much better you can access ARVs; you can access STI screening; your TB screening. All those things you can access them all around without having to go to the clinic… There is a staff that is LGBTQI+ or friendly so they get it that you can arrive as so and so with makeup and your lipstick and tell them that you are trans and show them your ID and your ID will still say male and they will get it.’ (Participant #2, 33 years old, educator)

Seeking healthcare support from NGOs is reinforced by the research of Sekoni et al.^12^ Despite good healthcare services provided by NGOs, the results also found a limited availability of these services as a barrier to accessing these services. Sherman et al.^6^ in their study on barriers to accessing and engaging in healthcare among black transgender women in the United States similarly observed a scarcity of healthcare providers specialising in gender-affirming care. Many participants in the study also reported struggling to reach these centres because of a lack of money for transportation, corroborating findings by Ogunbajo et al.^31^ in this regard.

#### Sub-theme 3: Using traditional healers

A few participants reported using traditional healers or medicine as an alternative to mainstream healthcare. Two participants reported that they received acceptable treatment from traditional healers and reported not being judged or rejected when using traditional medicine while being trans, HIV-positive and on ARVs. A comment from Participant 1 captured these sentiments in the following way:

‘It is the only place that we go to get help without being discriminated; I mean transgender women and traditional healers you know; traditional healers believe that transgender women there is a spirit that has come back in your body. It’s your ancestor of some sort and those types of things; so you would go there and get help.’ (Participant #1, 50 years old, transwomen advocate and lobbyist)

However, some participants expressed reluctance to use traditional medicine because of concerns that it could interfere with the effectiveness of ARVs.

### Strengths and limitations of the study

This was a pioneering study to investigate alternative HIV healthcare solutions among transgender women in South Africa. The findings offered valuable insights for programme planning, especially concerning gender-affirming healthcare and HIV/acquired immunodeficiency syndrome (AIDS).

The study also came with some limitations. It was conducted only in one municipal district, excluding other areas within the province. Moreover, the small sample size, with only 10 participants, also limits the generalisability of the findings.

### Implications for policy and practice

This study highlighted the challenges in accessing health services for transgender women in mainstream care. Moreover, transgender women were found to avoid health services in general, often because of concerns about stigma and discrimination. Considering these findings, decision-makers in healthcare facilities should improve services for transgender women. In the long term, it is recommended that gender-affirming healthcare services be made available in primary healthcare facilities, as these are more accessible to most transgender women. Providing gender-affirming care in primary healthcare facilities could also encourage this population to seek HIV prevention services more often. Changes to national, provincial and district healthcare policy frameworks, including amendments to certain health acts concerning the provision of gender-affirming healthcare in the context of HIV/AIDS, TB and STIs, may be necessary to facilitate the provision of these services.

The study showed that NGOs are key players in providing healthcare tailored to transgender women, including transitioning assistance, HIV prevention, treatment, psychological support and education. The limited availability of these services outside urban areas and transportation barriers to access these NGOs proved problematic. It would be beneficial to expand and support these services to ensure accessibility for all transgender women.

## Conclusion

The study aimed to assess the healthcare needs of transgender women, their experiences of the mainstream healthcare system and alternative strategies for navigating the healthcare system to meet their healthcare needs.

The main findings showed a desire for equitable access to healthcare services, specifically HRT, HIV treatment and prevention, and treatment for STDs. Accessing HRT posed a significant challenge because of limited availability in mainstream healthcare facilities.

Participants reported continuously encountering marginalisation and discriminatory treatment when attempting to access healthcare services within mainstream healthcare settings, characterised by experiences of trauma, hostility and considerable burden for these individuals. Despite these various challenges, transgender women exhibited resilience by employing alternative strategies to meet their healthcare needs.

Our study adds the following important new insights:

- It provided a first look into how and to what extent transwomen employ alternative healthcare strategies such as self-medication and utilising NGOs when faced with mainstream healthcare access barriers in a South African context.
- A novel, alternative strategy used by transwomen to access healthcare and treatment was identified in this study. Traditional doctors were found to be generally open to treating transgender women without discrimination and at a reasonable cost. The role that indigenous knowledge systems may play in the health management of transwomen warrants further investigation.^33^
- Healthcare professionals in South Africa must be equipped with the necessary know-how to provide adequate transgender healthcare. Increasing awareness, knowledge levels (particularly about HIV/AIDS and hormone intake) and positive attitudes of healthcare providers about transgender issues could encourage transwomen to utilise healthcare services more often.
